# *HPC2/ELAC2* polymorphisms and prostate cancer risk: analysis by age of onset of disease

**DOI:** 10.1038/sj.bjc.6600564

**Published:** 2002-10-07

**Authors:** J C Meitz, S M Edwards, D F Easton, A Murkin, A Ardern-Jones, R A Jackson, S Williams, D P Dearnaley, M R Stratton, R S Houlston, The Cancer Research UK/BPG UK Familial Prostate Cancer Study Collaborators, R A Eeles

**Affiliations:** Institute of Cancer Research, 15 Cotswold Road, Sutton, Surrey SM2 5NG, UK; Cancer Research UK Genetic Epidemiology Unit, Strangeways Research Laboratory, Worts Causeway, Cambridge CB1 8RN, UK; Data Management Unit, Translational Cancer Genetics Team, Institute of Cancer Research, 15 Cotswold Road, Sutton, Surrey SM2 5NG, UK; Royal Marsden NHS Trust, Downs Road, Sutton,Surrey SM2 5PT, UK (List of collaborators available on request)

**Keywords:** *ELAC2*, *HPC2*, prostate cancer (PrCa) susceptibility, polymorphisms

## Abstract

The candidate prostate cancer susceptibility gene *HPC2/ELAC2* has two common coding polymorphisms: (Ser→Leu 217) and (Ala→Thr 541). The Thr541 variant in the *HPC2/ELAC2* gene has previously been reported to be at an increased frequency in prostate cancer cases. To evaluate this hypothesis we genotyped 432 prostate cancer patients (including 262 patients diagnosed ⩽55 years) and 469 UK, population based control individuals with no family history of cancer. We found no significant difference in the frequencies of Thr541-containing genotypes between cases and controls (OR=1.41, 95% CI 0.79–2.50). The association remained non-significant when the analysis was restricted to cases divided by age of onset into those diagnosed ⩽55 years (OR=1.50, 95% CI 0.79–2.85) or to patients diagnosed >55 years (OR=1.27, 95% CI 0.59–2.74). We conclude that any association between the Thr541 variant and prostate cancer is likely to be weak.

*British Journal of Cancer* (2002) **87**, 905–908. doi:10.1038/sj.bjc.6600564 www.bjcancer.com

© 2002 Cancer Research UK

## 

The *HPC2/ELAC2* gene, located on chromosome 17p11.2 was recently mapped and characterised (Tavtigian *et al*, 2001). The *ELAC2* gene product has an amino acid sequence similarity to DNA interstrand crosslink repair proteins (PSO2, SNM1) and the 73-kD subunit of mRNA 3′ end cleavage and polyadenylation specificity factor (CPSF73). Tavtigian *et al* (2001) reported linkage to prostate cancer (PrCa) in this region, using a set of 33 extended multiple case pedigrees from Utah. They obtained a maximum two point lod score of 4.5 at marker D17S1289 (θ=0.07) and a three point lod score of 4.3 using the markers D17S1289 and D17S921 (θ=0.10) using a recessive model. Mutational analysis of *HPC2/ELAC2*, using an extended PrCa family set of 127 pedigrees with individual family lod scores of ⩾1 or >six cases sharing a haplotype, revealed that a subset of individuals in one family carried a germline frameshift mutation (1641insG). A common missense variant (serine to leucine at amino acid 217) was found in 13% of PrCa cases *vs* 9% of unaffected pedigree members and 6% of controls (Tavtigian *et al*, 2001). The authors also reported a second non conservative missense change in the *ELAC2* gene, alanine to threonine at amino acid 541, that lies at the amino border of the histidine motif in the *ELAC2* gene (Tavtigian *et al*, 2001). These two polymorphisms are in strong linkage disequilibrium, such that the Thr541 allele only occurs on a Leu217 background. The authors obtained a higher frequency of Thr541 carriers in PrCa cases in their families than in ‘divergent controls’ (unaffected spouses from other cancer families) (OR=3.1, *P*=0.022), though not when compared with unaffected individuals in the PrCa families. Tavtigian *et al* (2001) postulate that the Leu217/Thr541 haplotype results in more functional disruption of the protein than the commoner Leu217/Ala541 haplotype. An association between the Thr541 allele and PrCa was also reported by Rebbeck *et al* (2000), who estimated an odds ratio for PrCa of 2.37 (95% CI 1.06–5.29) associated with carrying the Thr541 allele. Among the 266 male control subjects (matched for age and race), a Thr541 allele frequency of 2.9% and a Leu217 allele frequency of 31.6% were reported. Suarez *et al* (2001) also found a significantly higher frequency of Thr541 alleles in PrCa cases than in controls (OR=3.6, 95% CI 1.8–7.3) in a study of 257 multiplex PrCa sibships and 355 controls. However, four recently published studies (Rökman *et al*, 2001; Vesprini *et al*, 2001; Wang *et al*, 2001; Xu *et al*, 2001) reported no evidence for an association between the Leu217 and Thr541 variants and PrCa risk. Xu *et al* (2001) also reported no linkage at *HPC2* after genotyping 159 hereditary PrCa families (with at least three affected family members) and analysing them using both parametric and nonparametric methods.

In an attempt to confirm or refute the association of *HPC2* and PrCa, and in addition to investigate any association with age of onset, we have genotyped these variants in a series of 432 PrCa patients from the UK, including 262 patients diagnosed ⩽55 years, and 469 controls.

## MATERIALS AND METHODS

### PrCa patients

This study was conducted on prostate cancer samples from patients treated in the UK. We obtained 432 blood samples from two series of PrCa Patients via consultants collaborating in the Cancer Research UK (formerly Cancer Research Campaign)/British Prostate Group (CRC/BPG) UK Prostate Familial Cancer Study (described by Eeles, 1999) and from the Royal Marsden NHS Trust (RMNHST) PrCa clinic. The PrCa cases were collected in two sets.

Series (I) A national study of early onset PrCa, selecting patients diagnosed at age ⩽55 years; patients were referred into this study through collaborating clinicians across the UK (The CRC/BPG Study). Patients were not selected on the basis of their family history. The average age of diagnosis of the PrCa patients was 51 years (range 24–55 years).

Series (II) A systematic series of PrCa cases, ascertained through the Urology Unit of the Royal Marsden Hospital, London, UK over the period 1992–1993. These patients were unselected for age or family history. The average age of diagnosis of the PrCa patients was 69 years (range 55–80 years).

In the analysis patients were subdivided into those diagnosed ⩽55 years (*n*=262) and those diagnosed >55 years (*n*=170). For the purpose of this analysis we excluded non-Caucasian patients;

### Controls

We had two control series available for analysis. Series (1) comprised spouses of patients enrolled in an UK population based study of colorectal cancer (number available=285; 136 female, 149 male; number informative=276). Of these informative controls, 142 were male and 134 were female. Series (2) was selected from the controls in an UK population based study of breast cancer diagnosed ⩽45 years age (number available=223, number informative=193;). The controls in series (2) were female. All controls were cancer free at the time of ascertainment. A total of 469 control samples were genotyped.

### DNA extraction

DNA was extracted from blood samples by routine methods with the inclusion of a second proteinase K digestion at 50°C. (Edwards *et al*, 1997). DNA was dissolved in 0.2 to 0.4 ml of water (BDH, Poole, UK) and stored at −20°C until required.

### PCR and genotyping

#### Ser217Leu variant

The PCR primers used were designed by Tavtigian *et al* (2001) and also published by Rebbeck *et al* (2000). The region containing the Ser217Leu variant was amplified as described by Rebbeck *et al* (2000) using a 65°–51°C touchdown protocol. The 335 bp PCR product (15 μl) was digested overnight with 15 U Taq I α (NEB RO149L) at 65°C. Genotypes were visualised on a 2.5% Agarose Gel (Metaphor Agarose). The PCR product sizes after digestion were either 173+162 bp, for the Ser/Ser genotype or 335 bp for the uncut Leu/Leu genotype or 335+173+162 bp for the Ser/Leu genotype.

#### Ala541Thr variant

The primers used to amplify the region of the Ala541Thr variant were described by Rebbeck *et al* (2000). The PCR product was digested overnight with 2 U Fnu4HI (NEB, R0178L) at 37°C. The digested 495 bp PCR product was visualised as either 250+110 bp fragment (Ala/Ala genotype) or as 250+162+110 bp (Ala/Thr genotype) always with three smaller fragments (34, 49, 52 bp). We did not observe any Thr/Thr genotypes (250+162+49+34 bp).

### Analysis

Analyses of genotype frequencies for each polymorphism were based on all cases and controls successfully typed for each polymorphisms, whereas analyses of combined genotypes at codons 217 and 541 were based on individuals successfully genotyped at both loci (Table 2

**Table 2:**
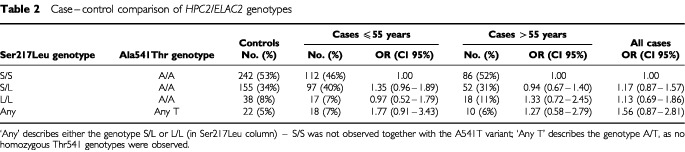
Case–control comparison of *HPC2/ELAC2* genotypes

), so the numbers of cases and controls differ slightly between analyses. Differences in genotype frequencies between cases and controls were tested using standard chi-squared tests. Odds ratios and confidence limits were calculated using standard methods. Trends in genotype frequencies by age at diagnosis were tested using logistic regression. The meta-analysis of genotypic risks over all studies was also conducted using logistic regression, treating each study as a separate stratum.

## RESULTS

The estimated allele frequencies in the combined control series were 2.4% for the Thr541 allele and 28.3% for the Leu217 allele (based on 457 controls, successfully genotyped for both polymorphisms). In agreement with previous studies we observed a Thr541 allele only in the presence of a Leu217 allele. The control frequency of the Leu217 allele (32 *vs* 26%) and of the Thr541 allele (3.4 *vs* 1.6%) was higher in series (2) than in series (1) controls. Neither difference was, however, statistically significant (χ^2^=2.28, *P*=0.13 and χ^2^=3.51, *P*=0.06) respectively and we therefore combined our control series for the main analysis. There was also no significant difference between the allele frequencies of males and females within control series (1).

To do the main calculations for the association between the Thr541 variant and PrCa risk, another 18 PrCa cases diagnosed ⩽55 years and also four PrCa cases diagnosed >55 years, that only failed for the Ser217Leu genotype were included in the analyses. We found no significant difference in the frequency of Thr541 containing genotypes between cases and controls (OR 1.41, 95% CI 0.79–2.50, Table 1

**Table 1:**
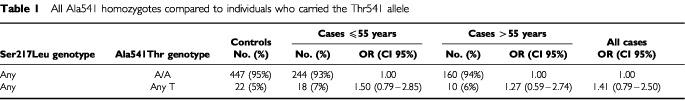
All Ala541 homozygotes compared to individuals who carried the Thr541 allele

). This remained true when attention was restricted to patients diagnosed ⩽55 years (OR 1.50, 95% CI 0.79–2.85) or to patients diagnosed at >55 years (OR 1.27, 95% CI 0.59–2.74). There were also no significant differences in the genotype frequencies of the Ser217Leu polymorphism. Estimated odds ratio for the genotypes based on both polymorphisms (Leu217/Thr541 genotypes; S/L/A/T, L/L/A/T) are shown in Table 2. The odds ratio for Thr541 carriers, compared with Ser217/Ala541 homozygotes was slightly elevated (1.56, 95% CI 0.87–2.81) but still not significantly different from 1.00. We also tested for a trend in Thr541 frequency with age at diagnosis, but found no significant effect.

## DISCUSSION

We attempted to replicate the association between Thr541 and PrCa risk reported by Tavtigian *et al* (2001) and Rebbeck *et al* (2000), but found no significant effect in our dataset. The estimated effect in our study (odds ratio 1.41) was in the same direction as the previous studies, but the magnitude of the effect was markedly smaller. However confidence limits on these odds ratios are still wide, and the results are still consistent with a two-fold increased risk.

We also analysed the data on the basis of combined Ser217Leu and Ala541Thr genotypes. We found a slightly higher odds ratio for Thr541 carriers compared with Ser217/Ala541 homozygotes (OR 1.56, 95% CI 0.87–2.81; Table 2) and compared with all other Ala541 homozygotes (OR 1.41, 95% CI 0.79–2.50, Table 1). This difference is a result of the slightly (but not significantly) higher frequency of Ser217/Leu217 carriers in PrCa cases, a trend which is more marked in cases diagnosed ⩽55 years (OR=1.35, 95% CI 0.96–1.89). This effect was also seen by Tavtigian *et al* (2001) and Rebbeck *et al* (2000). If these were true differences, they would imply a small PrCa risk associated with the Leu substitution and a larger risk associated with Thr541. However, the Leu217 effect is not significant in any study.

A notable feature of our study was the large proportion of cases (*n*=262) diagnosed at ⩽55 years of age, which we ascertained through an UK national study of early onset PrCa. The familial risk of PrCa is much higher at young ages (Carter *et al*, 1992) and one might therefore expect any effect of *HPC2* to be stronger in this age group. Moreover, screen detected disease is almost entirely absent in this group. The estimated odds ratio associated with Thr541 was higher in this age group, but it was still less than two and not significantly different from that in the older age group (OR 1.77, 95% CI 0.91–3.43 *vs* 1.27, 95% CI 0.58–2.79).

Suarez *et al* (2001) did not find a significant difference in the frequency of Ser217Leu variants between cases and controls (χ^2^=1.79; *P*=0.18) but found a significantly greater frequency of the Ala541Thr variant in cases than in controls (χ^2^=7.13; *P*=0.008); this gives an estimated OR of 3.6 (95% CI 1.8–7.3). The study genotyped 257 PrCa patients (multiplex sibships) and 355 race matched healthy unrelated controls. No excess clustering of the Thr541 allele was found within the multiplex families which suggested no evidence for linkage of PrCa to the *HPC2/ELAC2* gene.

Four other recent studies showed a negative risk association of *HPC2* with PrCa. Xu *et al* (2001) exclude a major contribution of *HPC2/ELAC2* as a high-prevalence, high-penetrance major gene for PrCa after performing linkage analysis in multiple case PrCa families and mutation screening (using DHPLC) of all coding exons of the gene in 93 probands. The study also genotyped 159 PrCa probands with a family history (hereditary prostate cancer, HPC), 249 PrCa patients with sporadic PrCa and 211 unaffected male control subjects. The results for Thr541 carrier frequencies were 10.5% for HPC patients, 9.0% for patients with sporadic PrCa and 9.0% for the control subjects. (HPC OR=1.37, 95% CI 0.61–3.11 for low risk allele S/S/A/A)

In another recently published study by Vesprini *et al* (2001), 431 screen detected PrCa patients with an elevated serum-PSA level (>4.0 ng ml^-1^) were genotyped for the two missense mutations Ser217Leu and Ala541Thr. Also 531 men, who underwent prostatic biopsy but had no evidence of invasive cancer were genotyped and were then used as male controls. Another control population consisted of 922 healthy, unselected women from the same population. The group found a similar Thr541allele frequency between 6.3 and 6.8% for all their patients and controls respectively, which lead to the conclusion that the Thr541 variant is unsuitable for use as a screening method for PrCa patients with a raised PSA. There was no association between the Ala541Thr variant and the risk of screen detected PrCa (OR 1.10; 95% CI 0.48–2.50; *P*=0.84).

Rökman *et al* (2001) genotyped 107 HPC cases, 467 unselected PrCa cases and 223 benign prostatic hyperplasia (BPH) cases and 568 healthy male blood donors for the two *HPC2* variants Leu217 and Thr541. The study found no difference in the frequencies of Leu217 and Thr541 in HPC patients, unselected PrCa patients or the control group (42–54% and 7.5%) respectively, but a slightly higher frequency of the Thr541 variant was observed in the BPH cases (OR=1.73, 95% CI 1.04–2.87). The group also performed mutation screening in the *HPC2/ELAC2* gene using SSCP and found the variant Glu622Val. The results showed an almost three-fold increased risk of PrCa in carriers of the Val622 mutation compared to non-carriers (OR=2.94, 95% CI 1.05–8.23).

Wang *et al* (2001) found no association of Ser217Leu and Ala541Thr with PrCa risk by genotyping 446 PrCa patients from 164 families and 502 population based controls (OR=1.04; 95% CI 0.57–1.89). The Leu217 frequency in PrCa patients was 32.3% and in controls 31.8%. The frequency of the Thr541 variant was 5.4% in patients *vs* 5.2% in controls.

In order to evaluate the overall evidence for an effect of the Ala541Thr polymorphism on PrCa risk, we performed a meta-analysis based on the results of the seven published studies (Rebbeck *et al*, 2000; Rökman *et al*, 2001; Suarez *et al*, 2001; Tavtigian *et al*, 2001; Vesprini *et al*, 2001; Wang *et al*, 2001; Xu *et al*, 2001), together with the current study (Table 3Table 3

**Table 3:**
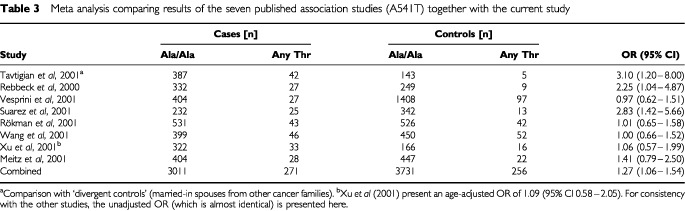
Meta analysis comparing results of the seven published association studies (A541T) together with the current study

). The estimated odds ratio for the Thr541 carriers compared with all other genotypes was 1.27 (95% CI 1.06–1.54, *P*=0.012). However, there is significant evidence of heterogeneity in the odds ratio estimates among the studies (χ_2_^7^=16.18, *P*=0.024). If the Vesprini *et al* (2001) data (which is based on screen detected cancers and controls undergoing screening) is excluded, the estimated odds ratio was 1.36 (95% CI 1.10–1.68, *P*=0.004). However, if the initial hypothesis-generating studies of Tavtigian *et al* (2001) and Rebbeck *et al* (2000) are also excluded, the evidence is much weaker (OR 1.21, 95% CI 0.96–1.53, *P*=0.10).

We conclude that the *HPC2* variants are associated with, at most, a moderate increased risk of PrCa. In particular Thr541 is unlikely to be associated with a risk of more than 1.5-fold.
